# Management and Quality Control of Large Neuroimaging Datasets: Developments From the Barcelonaβeta Brain Research Center

**DOI:** 10.3389/fnins.2021.633438

**Published:** 2021-04-15

**Authors:** Jordi Huguet, Carles Falcon, David Fusté, Sergi Girona, David Vicente, José Luis Molinuevo, Juan Domingo Gispert, Grégory Operto, for the ALFA Study

**Affiliations:** ^1^Barcelonabeta Brain Research Center, Barcelona, Spain; ^2^Barcelona Supercomputing Center, Barcelona, Spain

**Keywords:** processing workflows, neuroimaging, quality control, data management, neuroinformatics, cohort studies

## Abstract

Recent decades have witnessed an increasing number of large to very large imaging studies, prominently in the field of neurodegenerative diseases. The datasets collected during these studies form essential resources for the research aiming at new biomarkers. Collecting, hosting, managing, processing, or reviewing those datasets is typically achieved through a local neuroinformatics infrastructure. In particular for organizations with their own imaging equipment, setting up such a system is still a hard task, and relying on cloud-based solutions, albeit promising, is not always possible. This paper proposes a practical model guided by core principles including user involvement, lightweight footprint, modularity, reusability, and facilitated data sharing. This model is based on the experience from an 8-year-old research center managing cohort research programs on Alzheimer’s disease. Such a model gave rise to an ecosystem of tools aiming at improved quality control through seamless automatic processes combined with a variety of code libraries, command line tools, graphical user interfaces, and instant messaging applets. The present ecosystem was shaped around XNAT and is composed of independently reusable modules that are freely available on GitLab/GitHub. This paradigm is scalable to the general community of researchers working with large neuroimaging datasets.

## Introduction

Neuroimaging has now taken a central role in the context of research in Alzheimer’s disease (AD) as in neuroscience in general. Its non-invasive nature, its relative widespread availability, and its potential to provide efficient disease predictive markers have incentivized global efforts to assemble large imaging datasets, with numbers of subjects starting to reach ranges of epidemiological studies (Van Horn and Toga, 2014; Abe et al., 2015; Júlvez et al., 2016; Miller et al., 2016; Cox et al., 2019). With the advent of modern computational methods and the constant progress in imaging techniques, images are now routinely taken through automatic processing workflows, yielding a series of endpoints to be analyzed against other variables, which may potentially develop into findings. Despite good practices and quality assurance (QA), each step (acquisition or processing) is likely to exhibit anomalous behaviors and may lead to erroneous conclusions if unnoticed. In this regard, quality control (QC) protocols are designed to track down and protect against such errors but have until now faced major obstacles. Their purpose is to assess the conformity of any applicable dataset with a set of custom specifications and consequently determine whether the dataset is suited for further processing/analysis. On the one hand, individual visual inspection has proven to be neither fail-safe nor compatible with the size of the largest cohort studies (Alfaro-Almagro et al., 2018). On the other hand, automated or semi-automated QC offers promising cost-reducing perspectives (Esteban et al., 2019a; Sunderland et al., 2019); however, it remains hard to generalize as it strongly depends on the study design (single/multisite, clinical/cohort study) and needs to be adapted to each imaging sequence (Oguz et al., 2014; Bastiani et al., 2019) and each step of the workflow (raw images, processing outputs) (Klapwijk et al., 2019). Table 1 draws an inventory of existing resources focused on QC of neuroimaging data, automated or not, with corresponding references and repositories, if applicable. This list is first and foremost illustrative of their variety and specificity in relation to types of input data. Interestingly, the recent years have seen the emergence of new approaches aiming at unifying, on one side, QC protocols across groups and, on the other, processing workflows in some of these modalities such as structural magnetic resonance imaging (MRI) (Esteban et al., 2017) or functional MRI (Esteban et al., 2019b). Such approaches may pave the way for a general process of standardization of QC tools and procedures that would extend to most used neuroimaging data modalities.

**TABLE 1:** List of currently available resources intended for quality control of neuroimaging data (adapted from https://incf.github.io/niQC/tools).

Name	References	Data	Technology	Code repository
dashQC	n/a	fMRI, registration	Javascript	https://github.com/SIMEXP/dashQC_fmri/issues
qcApp	n/a	FreeSurfer	Java	https://github.com/ntraut/QCApp
qsiprep	n/a	DWI	Python	https://github.com/pennbbl/qsiprep
uniQC	n/a	fMRI	Matlab	https://github.com/CAIsr/uniQC
exploreDTI	Leemans et al., 2009	DWI	Matlab	n/a
dtiprep	Oguz et al., 2014	DWI	C++	https://github.com/NIRALUser/DTIPrep
PCP-QAP	Shehzad et al., 2015	T1w, fMRI	Python	https://github.com/preprocessed-connectomes-project/quality-assessment-protocol
brainbox	Heuer et al., 2016	segmentation	Javascript	https://github.com/OpenNeuroLab/BrainBox
exploreASL	Mutsaerts et al., 2017	ASL	Matlab	n/a
mriqc	Esteban et al., 2017, Esteban et al., 2019a	T1w, fMRI	Python	https://github.com/poldracklab/mriqc
PALS	Ito et al., 2018	T1w, fMRI	Python	https://github.com/npnl/pals
rtQC	Heunis et al., 2019	fMRI	Matlab	https://github.com/rtQC-group/rtQC
visualqc	Raamana, 2018	T1w, FreeSurfer	Python	https://github.com/raamana/visualqc
mindcontrol	Keshavan et al., 2018	FreeSurfer	Python, Javascript	https://github.com/OpenNeuroLab/mindcontrol
AFQ-Browser	Yeatman et al., 2018	DWI	Python, Javascript	https://github.com/yeatmanlab/AFQ-Browser
braindr (braindrles)	Keshavan et al., 2019	snapshots	Javascript	https://github.com/OpenNeuroLab/braindr; https://github.com/SwipesForScience/SwipesForScience
eddyqc/quad/squad	Bastiani et al., 2019	DWI	C (FSL)	https://git.fmrib.ox.ac.uk/matteob/eddy_qc_release
fmriprep	Esteban et al., 2019b	fMRI	Python	https://github.com/poldracklab/fmriprep
qoala-t	Klapwijk et al., 2019	FreeSurfer	R	https://github.com/Qoala-T/QC
snaprate	Operto, 2019	snapshots	Python, Javascript	https://github.com/xgrg/snaprate
nisnap	Operto and Huguet, 2020	snapshots	Python	https://github.com/xgrg/nisnap

lImproved data management is also directly associated with improved quality assessment: a system in which one can easily find and work with the data is likely to make quality assessment easier. Inversely, a system in which finding the data is complicated will make quality assessment much harder. As a consequence, the capacity to evaluate the results of any workflow and the capacity to identify/navigate through them in a larger repository are both tightly coupled. This is especially relevant for workflows such as the ones used in neuroimaging studies, which typically combine high levels of complexity, heterogeneity (e.g., in numbers of files, nature/structure of data) on the one hand, and, on the other, a high degree of required expertise to assess their outputs. With respect to this, to date, individual research groups may choose among different strategies, essentially based on their size and allocated resources, among which:

– organizing a local file repository and relying on core tools/libraries, predefined procedures and adoption of best practices.
– setting up a local management platform by building upon some existing open-source or proprietary systems (or developing it from scratch).
– subcontracting data management as a service, as included in “Science in the cloud” solutions.

Different sets of technical solutions exist for each of these approaches. In particular, initiatives such as BIDS (Gorgolewski et al., 2016) or BIDS-Apps (Gorgolewski et al., 2017) play an extremely valuable role in the spread of software-engineering practices along the neuroimaging research workflow, with beneficial consequences on reproducibility. The BIDS standard has become, over the past years, a spearhead in the promotion of FAIR principles (Wilkinson et al., 2016) by addressing data *findability*, *reusability*, and *interoperability* across groups, systems, and tools. As BIDS provides the formalism to organize the data and metadata, data *accessibility*, for its part, requires additional software that will generally include basic features for data management and exploration. As two open-source cloud-based solutions that have built upon BIDS, OpenNeuro (Poldrack et al., 2013) and Brainlife.io (Avesani et al., 2019) are iconic examples of platforms giving access not only to datasets but also to online computational resources, giving substance to the concept of virtual laboratory (Frisoni et al., 2011). As such, the purpose of the “Science in the cloud” model is also to facilitate data sharing and reproducibility by centralizing resources for data storage, management, computation, and QC in the neuroinformatics field. This model has begun to spread (Redolfi et al., 2015; Manjón and Coupé, 2016; Kiar et al., 2017; Glatard et al., 2018) and draws a promising future for the community. Notwithstanding the preceding, it may still fail to address immediate down-to-earth needs from small to average-sized research groups, especially the ones dealing with self-acquired imaging data. First, implementing these frameworks or adapting them locally requires strong IT skills and a specialized labor force, making it technically out of reach for many groups with insufficient human and/or computational resources, or without connection to large consortia. Second, relying on existing open-access instances is still hardly compatible with data confidentiality policies in most studies, as these are rarely permissive enough to allow upload to third-party platforms from the start. The basic needs of the many research groups include, for instance, basic data collection/querying/handling in average-sized datasets (e.g., up to several thousands of subjects), combined with further exploration/review along most typical analysis workflows. It is particularly compelling that in comparison to the magnitude of efforts underway to assemble large imaging datasets, the range of technical solutions to address such basic needs is actually limited. As previously reported by Nichols and Pohl (2015) and Shenkin et al. (2017), extensible neuroimaging archive toolkit (XNAT) (Marcus et al., 2007), LORIS (Das et al., 2010), and NIDB (Book et al., 2013) appear indeed as the main existing open-source neuroinformatics software platforms supporting data sharing.

Now that neuroscience has entered a propitious era of data and computation, practical solutions are still required to efficiently operate local databases and run tailored controls on complex type-agnostic raw and processed data.

Quality control and data management are thus both interrelated. They both have transversal impacts on the research workflow, from the data acquisition to the analysis. Both if poorly executed may have a strong negative impact on reproducibility. As advocated in the neuroimaging community, e.g., by the ReproNim initiative (Kennedy et al., 2019), core resources may already exist but their use should be facilitated so as reproducibility is achieved by design, not as an afterthought. Such considerations have nurtured the development of a novel infrastructure scheme–presented here–for imaging data management and processing, focused on facilitating scalable QC and aiming at maximizing the reuse of existing open core tools/libraries.

This model was implemented and adapted to the needs of a specific research program, namely, the ALFA project, yet with concerns about lean development principles and reusability. The ALFA project (Alzheimer’s and Families) is a research platform started by the Barcelonaβeta Brain Research Center (BBRC) for the prospective follow-up of a cohort of cognitively normal subjects–most of which are the offspring of AD patients. Extensive phenotyping of participants includes cognitive assessment, lifestyle questionnaires, blood extraction for further genetic analysis, cerebrospinal fluid collection, positron emission tomography (PET) imaging, and multimodal MRI examination performed on-site on a single Philips Ingenia CX 3T scanner. The interested reader may refer to Molinuevo et al. (2016) for a full description of the various arms of the project and administered examinations. Since 2012 when BBRC was created, its neuroimaging platform has been acquiring and is currently managing data from over 5000 participants across its different studies. Imaging protocols include standard MRI sequences (with T1/T2/diffusion-weighted, inversion recovery, and resting-state functional MRI), some more advanced ones (arterial spin labeling, susceptibility-weighted imaging, and quantitative flow, among others), and, for a subset of participants, PET imaging–fluorodeoxyglucose (FDG) and flutemetamol. This paper documents the core concepts and implementation of this infrastructure for imaging data management, processing, and control. The first section will detail the routine data flow at BBRC, which this infrastructure partially supports. In a second section, the paper will describe the different ways provided to researchers of the group to interact with the platform. The third section will focus on QC performed on large imaging datasets. The fourth section will then elaborate on the employed strategy to foster sustainability and reproducibility and describe principles for future development.

## BBRC: Anatomy of a Single-Site Imaging Research Platform

**FIGURE 1:**
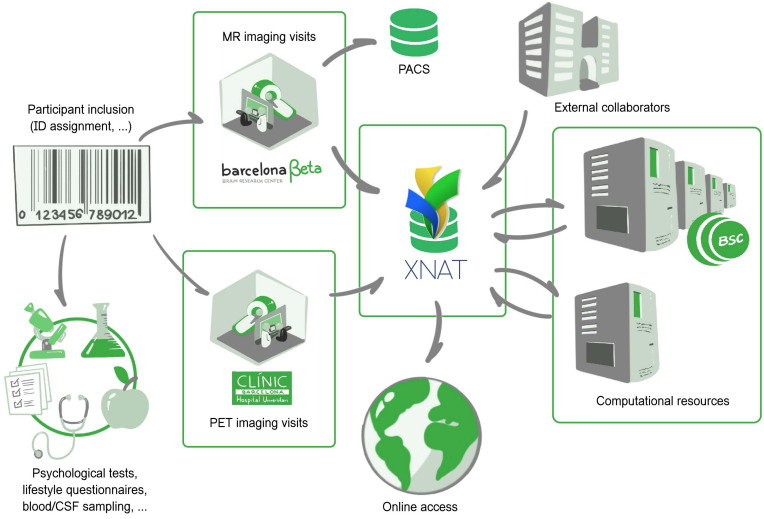
The Barcelonaβeta Brain Research Center: general view of the imaging data flow, from patient inclusion to data sharing. Imaging and non-imaging data follow different data flows. Imaging sessions are automatically imported in XNAT from the in-house MR scanner and external PET camera. Processing workflows are sent to computational resources from the Barcelona Supercomputing Center.

Participants may be included in one of the hosted programs such as the ALFA study, and get assigned with a unique accession number (Figure 1). This accession number is represented as a barcode and follows the participant through the whole acquisition protocol, which, on a standard basis, includes full neuropsychological evaluation, assessment of clinical history, APOE genotyping, lifestyle questionnaires, blood sampling, and–for a subset of individuals–cerebrospinal fluid extraction. Structural and functional MRI is acquired on-site on a dedicated MR scanner. Participants of the ALFA+ program undergo both flutemetamol and FDG PET at the Hospital Clinic of Barcelona.

Imaging and non-imaging data are stored and managed in two individual platforms. Non-imaging data are imported into a relational database and follow a specific data flow that is not described here. Imaging data are directly transferred from the scanner to both a PACS archive and an XNAT platform. Extensible neuroimaging archive toolkit (XNAT) (Marcus et al., 2007) is the most broadly deployed open source system to have emerged among imaging platforms in recent history. In this context, the PACS archive is used for long-term backup purposes, preserving a pristine copy of the acquired imaging data, and for daily routine visual review and reporting by radiologists, whereas XNAT is a much more flexible system geared toward researchers, allowing transformation, automatic processing, browsing, downloading, and eventually sharing. A Clinical Trial Processor (CTP) service (Aryanto et al., 2012) is run between the MR scanner and XNAT to ensure proper de-identification of protected health information. Outsourced PET imaging data are directly pulled from the acquisition site: a daily daemon service pulls new imaging scans from an sFTP server and pushes them to the PACS archive which then auto-forward to XNAT (via CTP). The workflow is open to external collaborators, who may also push data in independently managed projects distinct from the ALFA study.

Once the data have been successfully imported into XNAT, imaging sessions are routed to their corresponding XNAT project/study and then taken through automatic workflows. These workflows are managed by the XNAT Pipeline Engine, which directly draws computational power from the Barcelona Supercomputing Center^1^. Workflows include processing–e.g., involving all types of neuroimaging software or published methods/algorithms–but also automatic controls based on *Validators*, as described further in section “Generalized Automatic Sanity Check/Quality Control.” This results in the generation of derived images, numerical endpoints, or validation reports. Along with the primary raw data, they form the body of online available resources that users may reach by then logging into the system.

This data flow is presented in Figure 1.

## Better Control on Data by Providing Multiple Access Ways

### XNAT as the Infrastructure Core Engine for Imaging Data Management

Among the most significant ones from the last decade, neuroimaging projects like the Open Access Series of Imaging Studies (Marcus et al., 2010), IMAGEN (Schumann et al., 2010), the Human Connectome Project (Marcus et al., 2011), the International Neuroimaging Data-sharing Initiative (Mennes et al., 2013; Kennedy et al., 2016), the Adolescent Brain Cognitive Development (Casey et al., 2018), the UK Biobank (Miller et al., 2016), followed by the more recent ONDRI (Scott et al., 2020) or EPAD (Ritchie et al., 2020), have all in common that their respective infrastructures for data sharing are based on XNAT. This not only confirms the status of XNAT as a central technology but also highlights the opportunity of any model built around XNAT in terms of reusability.

We chose to rely on XNAT as the core engine of our infrastructure for imaging data. Among the few existing options available, XNAT offers an adequate cost–benefit ratio for groups of all sizes when comparing the complexity of implementation to all of its built-in features. XNAT provides tools for common management, user access, data processing, and sharing, thus covering many aspects of the basic neuroimaging workflow. It also includes a DICOM storage service (C-STORE SCP) for receiving and sorting images from any DICOM-compliant imaging device, which is essential for organizations managing their own imaging equipment. User access to the archive is provided by a secure web application. Workflow execution is enabled by a Pipeline Engine, while XNAT maintains full histories by tracking all changes to the data, thus enforcing data traceability. Finally, XNAT implements a security system that allows administrators to grant access to specific actions or datasets following predefined user roles.

To date, XNAT is still under active development with strong community-based support, aligning with current trends in the community as shown by recent support for BIDS format and containerized data processing (e.g., using Merkel, 2014). Most users may operate the database and search the repository through the built-in web-based application. Aside from this graphical interface, XNAT provides a Representational State Transfer (REST) Application-Program Interface (API) that allows users to query the database and therefore programmatic interaction with its contents. Furthermore, the *pyxnat* (Schwartz et al., 2012) library capitalizes on this API and allows users to interact with XNAT using Python.

We advocate that users should have multiple proposed ways and be free to choose their preferred one to operate the platform, as a greater flexibility in this regard is a stepping stone for improved data review and issue tracking. With respect to this, a few previous examples have built onto XNAT (Gee et al., 2010; Harrigan et al., 2016; Job et al., 2017), often leveraging its RESTful API (Schwartz et al., 2012; Gutman et al., 2014), to extend its standard features and present new ones. Such an approach stands out by its light footprint, relying on XNAT’s core features without needing to touch its codebase, to the mutual benefits of maintainability, dependability, portability, and usability. In line with this approach, this present paper describes a collection of lightweight solutions which together form an adaptive modular ecosystem focused on user experience and neuroimaging data QC.

### Barcelonaβeta + XNAT: *bx*

Interacting with the data on XNAT can be done mainly in two ways: either graphically using the web application or through a REST API. While the former is suited for all profiles, the latter is intended for a more technical category of users, allowing them to automate bulk operations, e.g., downloading large collections of data and populating projects or any type of systematic task that would otherwise, using the web application, require many manual operations. Version 1.7.5 of XNAT now includes a Desktop Client that may be used to download collections of images for instance from an entire study (or *project* in XNAT jargon). Still, between “all clicks” and “all script” lies a large gray zone with users who without being experienced coders may still have some knowledge on how to use command-line tools. For this special category, we wrote *bx*, which allows us to run from a terminal among a predefined set of bulk operations using a single command. This includes, for instance:

- downloading images of a given sequence over a project in the NIfTI format (better suited to a majority of post-processing software suites).
- downloading processing outputs over a project (e.g., segmentation maps, 3D models, etc.).
- downloading an Excel table with all numeric outcomes from a given pipeline over a project.
- downloading a table with acquisition dates from an entire project.
- in general, downloading any given type of resources over an entire project.

In particular, to get a local copy of the results from FreeSurfer *recon-all* pipeline (Fischl, 2012) over the entire XNAT project *ALFA*, one would simply run:

bx freesurfer6 files ALFA

Destination folder is set in a locally stored configuration file along with the user’s XNAT login credentials.

By extension, the following command:

bx freesurfer6 aseg ALFA

would generate a single spreadsheet file containing all the structural volumes estimated by FreeSurfer (in aseg.stats files). The current version (0.1.6) also includes, among others, commands for SPM (Ashburner and Friston, 2005), ANTs (Avants et al., 2009), FSL (Jenkinson et al., 2012), ASHS (Yushkevich et al., 2015), and CAT (Gaser, 2016), with subcommands for collecting output files, measurements yielded by the pipeline, QC-oriented snapshots, validation reports, or automatic test outcomes (as described later in section ‘‘Generalized Automatic Sanity Check/Quality Control’’). Importantly, any command may be applied to an entire project, one single MRI session, or also curated image collections^2^ relying on discretionary criteria (e.g., based on clinical, genetic or cognitive characterization, or any other external variable).

Such a tool thus provides an additional command-based way to interact with the XNAT data which optimizes a set of “frequent” use cases (based on user reports, like bulk downloading pipeline outputs) while abstracting the rest (i.e., obviating intermediate steps such as selection of subjects/experiments/resources). Since it was built over *pyxnat*, this makes it rather easy to get adapted to specific local configurations (or additional resources).

It is distributed as a PyPI package under the name *bbrc-bx* and hosted on GitLab: https://gitlab.com/xgrg/bx.

### Cron Jobs, Bots, and Monitors

**FIGURE 2:**
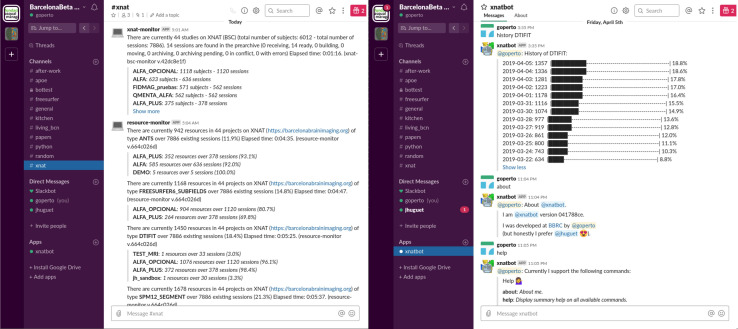
Screenshots of the #xnat channel from the Barcelonaβeta Slack workspace. **(Left)** Monitors provide members of the channel with daily updates on the current data available on the imaging platform without any user action. **(Right)** Basic human chatbot interactions give access to more specific statistics. In this example, the user is querying for the progress over time of some processing task (with DTIFIT).

In addition to *bx*-like scripts and XNAT’s standard interface, daily summaries are delivered automatically through both emails and instant messaging (IM). We built onto XNAT email notification service so that subscribed users receive a comprehensive sanity report (detailed in section “Generalized Automatic Sanity Check/Quality Control”) for every new session uploaded from the scanner. In parallel, automatic monitors running on a Slack (Johnson, 2018) #xnat channel provides authorized users with daily updates on numbers of available subjects/raw sessions per project and available resources such as processing outputs (derivatives) (left part in Figure 2). Such automatic delivery systems complement standard user experience by directly feeding with periodic statistics on the database, thus allowing to check instantly on the system’s general integrity status without user action. Users may also get further customized views on this information through basic human-chatbot interactions, e.g., longitudinal statistics. Figure 2 illustrates this integration: on the left, members of the #xnat channel are updated every day on available data, and on the right, users may ask about the progress over time (daily numbers of a given resource) of any pipeline on the platform. This approach may naturally be adapted to other messaging systems (e.g., Mattermost, Riot, Zulip, IRC) or project management tools possessing an API (e.g., Trello, Basecamp).

We advocate for giving users multiple controlled ways to deal with data. XNAT RESTful API is one of the most powerful features of its framework and allows to build a variety of access modalities, each of which comes with pros and cons. For example, the graphical user interface gives individual and comprehensive control on the data, though manually operated; *pyxnat* adds a programmatic interface to it and is, therefore, rather developer-oriented; *bx* optimizes bulk downloading operations from scripts, yet for a set of pre-selected resources; and IM-based tools provide only high-level summarized information but add an interactive and collaborative touch and nicely intertwine with natural conversations among users.

## “Given Enough Eyeballs, All Glitches Are Shallow”^3^

Each step of an analysis workflow should ideally be paired with specific checkpoints. Given the increasing quantity and complexity of datasets, relying on automatic control is imperative, but manual inspection can rarely be avoided. The following approach aims at capitalizing on automatic controls while allowing multiple users to jointly participate in visual inspection.

### Generalized Automatic Sanity Check/QC

**FIGURE 3:**
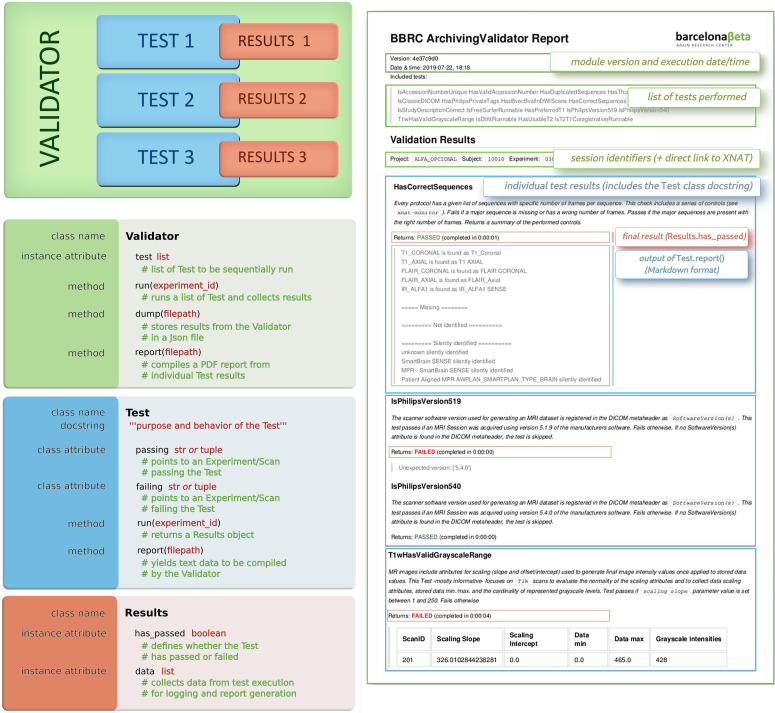
**(Left)** *Validators*: concepts and classes. *Validators* and *Tests* all share the same template. *Validators* are defined by a list of *Tests*, which in turn yield some *Results*. Each *Results* object embeds a main Boolean, which defines whether the *Test* was successful, and some additional data for logging purposes or report generation. **(Right)** Example of a produced validation report (only the first page is displayed); the color code highlights the matching between sections of the report and the corresponding concepts: green refers to *Validators*, each blue area corresponds to a *Test*, and *Results* are shown in red squares.

In line with recent trending standards such as BIDS (Gorgolewski et al., 2016) or NIDM (Keator et al., 2013), we present a validator-based modular approach, which, in our current implementation, covers image types such as T1-weighted, DWI, and PET images, and processing outputs like FreeSurfer, SPM, and FSL DTIFIT, and the approach may be easily extended to others. For each type of data, we define a tailored procedure for QC. Such procedures consist of predefined sequences of checkpoints: each checkpoint (later referred to as *Test*) is associated with some particular aspect of the data and would result either as passed or failed. In this present implementation, every new imaging resource pushed into the system is thus automatically taken through a QC procedure adapted to the type of data. Checkpoints are defined based on aspects of the data or metadata known to potentially exhibit undesired variability, e.g., due to technical or human-related factors. They may, for instance, include verifying that the output of some process matches some expected list of files, that some image parameters fall in specific intervals. Nevertheless, the approach is designed so as to give the most flexibility and scalability to the range of possible checkpoints. The use of a single template for all checkpoints–each of them being documented with human-readable specifications (e.g., detailed in each *Test*’s *docstring*, as explained hereafter) and resulting in a binary outcome–makes them easier to read and comprehend, especially in code. As a result, every new imaging session is provided with a checklist, by which the execution of further pipelines may be conditioned. It is worth noting though that by being designed as an independent command-line tool, any procedure from this module may be executed, not only automatically, but also manually upon request on any applicable dataset. The tool, written in Python, is based on two nested concepts: *Tests* and *Validators* (Figure 3).

A *Validator* is an object defined by a set of *Test* objects, each of which would check specific traits of a given XNAT entity (e.g., an incoming imaging session, or results from a processing workflow). *Validators* are run like any other pipelines by XNAT Pipeline Engine, triggered by some functional events (e.g., archiving of a session and completion of a processing pipeline, among others). The outputs from these series of checks are stored as additional resources and would be used to infer, either by visual review or programmatically, on the validity of the target resource.

A *Test* is defined by a *run()* and a *report()* function. The *run()* function returns a *Results()* object that has two attributes, namely, *has_passed* (Boolean) and *data* (list). This *run()* function may target any resource, either an Experiment or a Scan (following the XNAT terminology). Every *Test* has also two hardcoded class-level attributes, namely, *passing* and *failing*, pointing at two Experiments (or two 2-uples Experiment + Scan) from the running XNAT instance on which the test should respectively pass and fail [used for continuous integration (CI)]. Depending on the test purpose, it may return *Results*(*has_passed* = True) or *Results*(*has_passed* = False). One additional *data* argument may be passed to the *Results* constructor to record extra information (e.g., elapsed time) from the test execution.

In practice, running a *Validator* on a given experiment takes its associated set of *Tests* and runs them sequentially. A *Test* may apply to a Scan instead of an Experiment (e.g., *checking that DICOM files have been converted to NIfTI*), in which case the *Test* could be performed over all the existing Scans of the Experiment. Upon failure of a *Test*, scan quality flags may be adjusted from usable to questionable/not usable on XNAT. Once completed, the *Validator* dumps the results data in a JSON record and generates a Markdown-based PDF report (Figure 3). This report is built by calling each *Test*’s *report()* function consecutively and compiling their results in as many individual sections. By default, every section includes the *docstring* attribute taken from every *Test* class for the sake of traceability and self-sufficiency.

Both resulting PDF and JSON files appear on XNAT as resources of the validated experiment, so that users may query on them^4^ or dump them from the entire database, e.g., into a single spreadsheet file^5^. This is made directly possible using *bx* commands (section “Barcelonaβeta + XNAT: *bx*”) thanks to the seamless integration between both tools.

One key strength of this model is its adaptability/genericity. It allows rapid implementation of new *Tests* on any type of imaging data provided it can be identified as an XNAT Experiment or Scan. The actual performed verifications are stated in the *run()* function and may hence use any required external library. Another key advantage is the low cost associated with CI-related maintenance. Regression testing is indeed critical for the system to be sustainable as more checkpoints and more data are added. Automated unit testing for CI is performed after every new change in the code, based on the two class attributes *passing* and *failing* provided for each *Test*. Every single *Test* is thus systematically re-executed against two specific cases after any change in the code. Along with this, each generated report includes a reference to the last SHA identifier issued by the version control system. As all Tests share the same template, the testing code for CI requires no updates and remains always adapted to any newly added *Test*. Such a design yields to a unit-test-to-production-code ratio currently under 1:30.

In our current implementation, *Tests* have so far covered aspects related to both MR and PET acquisition and their post-processing derivatives. Supplementary Table 1 gives an illustrative summary of currently implemented *Tests*, including their associated docstrings to describe their purpose.

For example, every time a new PET session is imported to XNAT, a *PetSessionValidator* is triggered. This *Validator* currently includes a set of nine Tests. The first one, *IsTracerCorrect*, checks that the tracer information is correctly registered in the DICOM headers. The second one, *IsSeriesDescriptionConsistent*, makes sure that metadata are consistent across the session; then, *IsScannerVersionCorrect* checks in the DICOM headers that the scanner model matches, in this case, “SIEMENS Biograph64 VG51C”. Then, follow *IsSubjectWeightConsistent* and *IsTracerDoseConsistent* controlling that the values registered for subject’s weight and tracer dose match some target intervals (between 40 and 150 kg and between 1.5e8 and 3.5e8 Bq, respectively). Finally, the *Validator* runs *IsSubjectIdCorrect* to ensure the subject’s ID has the right format; *HasUsableT1*, which checks whether the subject has a valid T1-weighted image stored on XNAT; and both *IsCentiloidRunnable* and *IsFDGQuantificationRunnable*, which assess whether the data are suited for the execution of two quantification pipelines.

**FIGURE 4:**
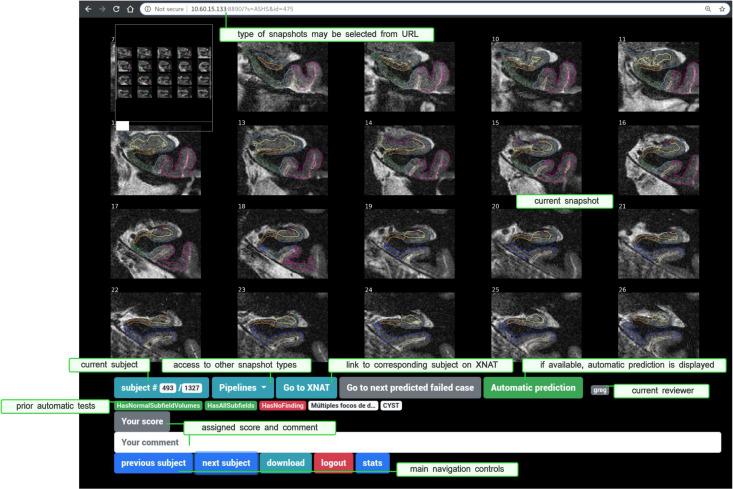
Snaprate: General user interface, running in a web browser. The upper part displays a zoomable snapshot (here a segmentation of hippocampal subfields). The lower part shows a section for the review section and navigation controls, including links to XNAT and to other types of snapshots. Results from prior checkpoints are also displayed in red (if failed) or green (if passed).

Another example is *ASHSValidator*, which is triggered every time some hippocampal subfield segmentation is executed over an MR session (using the ASHS pipeline). The *Validator* sequentially runs *HasAllSubfields*, which makes sure that all expected subfields appear in the final segmentation; *HasCorrectASHSVersion* controlling the software version; *HasCorrectItems* checking that the list of generated files matches the right one; *HasNormalSubfieldVolumes*, which assesses whether resulting subfield volumes fall inside some safety intervals; and *ASHSSnapshot*, which generates a snapshot of the final segmentation (shown in Figure 4).

Other *Validators* include, for instance, *ArchivingValidator* (triggered every time an MR session is imported/archived), *SPM12Validator*, *CAT12Validator*, *FreeSurfer6Validator*, *ANTSValidator*, and *DTIFITValidator* (triggered after every execution of SPM12, CAT12, FreeSurfer6, ANTS, and FSL DTIFIT, respectively). For a more comprehensive list of *Tests*, *Validators*, and details on their purpose, the reader may refer either to Supplementary Table 1 or directly to the code repository for the latest version, as sharing the same template [where each *Test* is a class with two test cases, a docstring, a *run()*, and a *report()* function, as described above] makes them easily readable.

The source code is released as an independent tool, *bbrc-validator*, available as a PyPI package and code is hosted on GitLab^6^.

### Generating Summarized Representations of Segmentation Results: *nisnap*

Among the broad typology of outputs generated by most neuroimaging analysis workflows, numeric and image-based results are probably the most common. In particular, any segmentation technique will generally yield either a label volume or probability maps to describe some target structures/objects, possibly coming with some derived numeric descriptors, as this is the case with standard software such as SPM or FreeSurfer for cortical/subcortical segmentation. Despite some recent efforts to predict it automatically (Klapwijk et al., 2019; Robinson et al., 2019), the assessment of their performance is still relying mostly on visual inspection. Pre-rendering summarized representations of these results, or *snapshots*, instead of any manual procedure involving standard visualization software (e.g., freeview, fsleyes, BrainVisa/Anatomist, and mricron) is a way to minimize time costs and risks of errors. To ease their generation from any Python-enabled environment, we released *nisnap* (Operto and Huguet, 2020). Through one main *plot_segment()* function, it includes controls for opacity, layout, color map, plane/slice selection, label picking, static, or animated rendering. Users may also choose between contours or solid color rendering. Though it also features a specific submodule for XNAT integration, *nisnap* is designed to be used with any individual NIfTI images. The function compiles a figure made of a selected set of slices, both from the input segmentation and (if provided) the original image, and renders an overlay of the former over the latter with the desired options. Animated mode generates a GIF animation with a fading effect on the segmentation. Eventually, an image file is created at the specified location with the resulting snapshot.

The tool may be used from Python scripts or command-line interfaces for offscreen rendering or from Jupyter notebooks for real-time visualization. In our context, *Validators* rely on *nisnap* to convert results from SPM, FreeSurfer, or ASHS into snapshots which are then included in validation reports (section “Generalized Automatic Sanity Check/Quality Control”). Snapshots are then collected in a subsequent step for visual review using *snaprate* (section “Assisted Visual QC: *snaprate*”). Figure 4 shows an example of snapshot produced by *nisnap* and displayed for review through *snaprate*.

*nisnap* is released as an independent tool, available as a PyPI package and code is hosted on GitHub^7^.

### Assisted Visual QC: *snaprate*

Automatic controls performed by *Validators* include generation of snapshots (e.g., for segmentation results using SPM, CAT, FreeSurfer, processing of diffusion-weighted imaging data using FSL, and registration using ANTs, among others). Although navigation is not enabled as it would be with a full-featured NIfTI viewer, e.g., Papaya^8^, brainbrowser (Sherif et al., 2015), and brainbox (Heuer et al., 2016), snapshots are lightweight resources that are displayed instantly and easily cacheable at runtime, hence resulting in optimized overall time of review. Such rendered representations allow fine-grained customization and are suited for the review of large collections of data. Nevertheless, they can still not be checked in a fully automatic way and generally require visual inspection. In particular, such an approach involving tool-assisted visual review of summarized versions of processing results has already been proposed, e.g., based on MR slices (Raamana, 2018) or pre-generated snapshots (Keshavan et al., 2019). Some alternatives include features for real-time NIfTI visualization and manual voxel labeling, thus enabling crowdsourced reviews and corrections (Heuer et al., 2016; Keshavan et al., 2018).

In line with this–and in order to minimize the burden given to experts and optimize the review process–we present an assisting tool (Figure 4) that naturally connects to the previously described system, collects previously generated snapshots (along with an optional predefined set of useful *Test* outputs), and displays them within a multi-user collaborative web application. Registered raters may navigate and assign each of them with a descriptive comment and a quality score. Snapshots are produced prior to the review process during automatic individual report generation, described in the previous section. Rendering is done based on either *nilearn.plotting* submodule (Abraham et al., 2014) or *nisnap* (as described in section “Generating Summarized Representations of Segmentation Results: *nisnap*”).

As snapshots are generated during the execution of Validators and their corresponding Tests, they may then be displayed along with the outcomes from those prior checkpoints. For instance, segmentation results produced by SPM12 come with prior Tests such as HasNormalVolumes (“do global gray/white matter volumes fall inside predefined target intervals?”) or SPM12SegmentExecutionTime (“did the pipeline take longer than a given threshold?”). Such checkpoints may be displayed under the snapshot to provide additional assistance to the review process. One of them can be selected, at the user’s choice, so that the navigation will jump from one failed case to the following one. In case further inspection of a given case is required, a direct link takes the user to the corresponding experiment on the XNAT platform. Users are also allowed to switch between pipelines/types of snapshots to assess their quality over the same subject (Figure 4).

We present *snaprate* (Operto, 2019) in its particular XNAT-centric software ecosystem. Nevertheless, the tool itself is designed to work alone with any type of pre-generated snapshots or figures. Here, image-based processing outputs are represented as a collection of slices either from the original images (e.g., fractional anisotropy or tensor maps from FSL DTIFIT) or from the original T1-weighted images overlaid with the segmentation/registration results (e.g., from SPM, CAT, FreeSurfer, ASHS, ANTs) (Figure 4). Prior to the review, all snapshots are extracted from reports and bulk downloaded into a single folder using *bx*^9^. Then, *snaprate* operates as a web application (using the *Tornado*^10^ Python web framework) on which users may log in using their individual browser. Every action (addition/edit of any score/comment) is automatically stored server-side as tabular data and may also be downloaded locally as spreadsheet files.

Code is available on GitHub at: http://github.com/xgrg/snaprate and a full demo can be found at http://snaprate.herokuapp.com.

## Discussion

Recent decades have witnessed an increasing number of large to very large imaging studies, prominently in the field of neurodegenerative diseases. The datasets collected during these studies form essential resources for the research aiming at new biomarkers. Nevertheless, setting up a basic infrastructure to collect, host, manage, process, review, and share those datasets is still a hard task, especially for organizations with their own imaging equipment, and the number of options in terms of existing open-source software platforms for neuroinformatics facilitating the seamless connection of an imaging scanner is still quite limited. Larger projects may afford to develop their own systems to serve these datasets, hence providing high-performance and customized service (e.g., primary access to the data, to computational resources, algorithms) to a restricted set of users. However, such systems are rarely designed to provide reusable solutions that could be easily adapted elsewhere. As opposed to this, the approach described in this article is characterized by its low footprint and high modularity, hence facilitating selective reuse and allowing incremental development. By low footprint, we suggest that the presented components not only introduce little dependencies (i.e., essential Python libraries) but also work with basic human-friendly objects (e.g., spreadsheets, JSON files, JPEG images, and PDF documents) making them again easily reusable independently.

The approach was implemented and is currently running in the context of an individual research institution managing cohort programs on risk factors and biomarkers of AD: the BBRC. It may in itself serve as a practical example for organizations with similar purposes. Such an empirical description, though, may not substitute a proper comparative study, not presented in this article, to assess the relative performance of this model. Nevertheless, it was built following guiding principles taken from best coding practices and software quality (e.g., extensibility, reusability, minimum cost to develop, clear definition of purpose) (Hoare, 1972). In that regard, all described components (*bx*, *nisnap*, *snaprate*, *bbrc-validator*) include diligent automated testing for CI (e.g., through sandboxed executions of most commands), thus yielding code coverage rates consistently over 90%. Additionally, as described in section “Generalized Automatic Sanity Check/Quality Control,” each *Test* in every *Validator* is, by definition, assigned with two imaging sessions, one that is expected to pass and the other, to fail. This not only complements the *Test*’s documentation by providing the reader with genuine examples but also ensures that *Tests* are systematically *tested* against real-life cases after every new change in the code. It is also worth noting that those current *Validators* (as the ones featured in Supplementary Table 1) have been tailored to the needs of one specific organization (e.g., checking the software version of a Philips MR scanner) and may be considered neither comprehensive nor suited for other institutions. However, the modularity and flexibility of the system allow them to easily adapt them to their respective contexts.

Another potential limitation of this present model is that by mostly focusing on automatic outputs, it is not well-adapted to handle manual corrections. In this version, workflows are automatically launched and managed through the XNAT Pipeline Engine, and their history is stored and searchable in the XNAT database. Pipelines are defined by a set of dependencies and conditions based on other pipelines and prior automatic tests. Failing cases are then flagged and ignored in subsequent steps. One drawback of this conservative approach is that failed cases (failed workflows or QC) are simply discarded from further analysis, resulting currently in a line loss of data that could probably be harnessed if processed manually. On the other hand, this strategy, by limiting manually input data/parameters, avoids the creation of forks and makes traceability easier to control by guaranteeing that any resource can only have a linear history. In this respect, coupling the system to a solution like DataLad (Wagner et al., 2019) to address version control may provide an interesting avenue for improvement.

**FIGURE 5:**
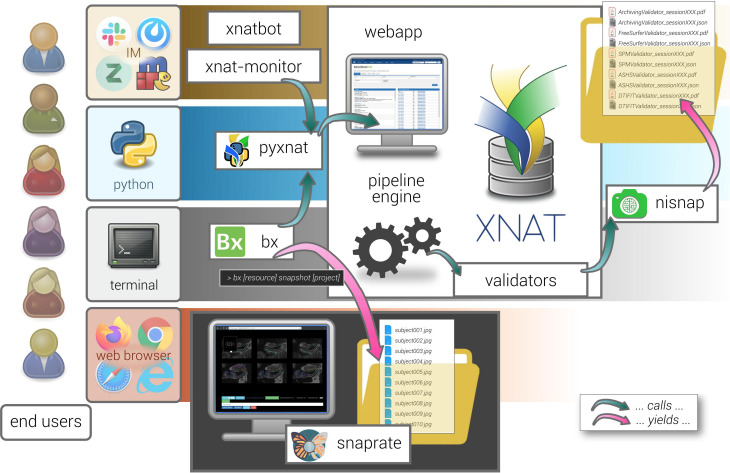
General view on the XNAT-based ecosystem architecture. The different satellite tools described in this manuscript are represented with their mutual interactions. Each of them is based on a specific type of user interaction, e.g., command line, scripts, web browser, and instant messaging (IM). Interaction with XNAT (e.g., xnatbot, xnat-monitor, bx, nisnap) relies on the pyxnat library. *Validators* are run as pipelines and produce reports (calling nisnap for snapshot generation). Snapshots are collected from any given XNAT project thanks to one of bx’s commands (*snapshot*) and passed to snaprate for visual quality assessment.

The overall system is built around XNAT, which is among the most broadly deployed open source systems for managing medical imaging data in research (Nichols and Pohl, 2015). We then enriched the platform with QC-oriented features by taking advantage of its REST API using Python (Schwartz et al., 2012). QC is balanced between automatic tests and tool-assisted visual inspection. On the one hand, automatic operations include sanity checks, collection of quality metrics, quality prediction, and generation of human-readable reports, all part of a single module, *bbrc-validator*, which was designed to have new tests easily added (and covered by CI automated testing). On the other hand, visual inspection is based on collaborative review of pre-rendered snapshots. Figure 5 illustrates this XNAT-centered ecosystem as a whole.

With all neuroimaging studies growing in scale and complexity, QA/QC has become a difficult task and a heavy burden, which is managed in very heterogeneous ways across research groups (depending on data type, sample size, experience, and resource availability, among others). Emergence of standardized QC methods is still required and is currently hindered by the existing variety of acquisition protocols (modalities and scanner manufacturers) or processing pipelines. While efforts have been initiated by the community in this regard–e.g., by the INCF Special Interest Group on Neuroimaging Quality Control^11^ (niQC)–common frameworks remain needed to make QC-related tasks easier and more efficient, with enough practical flexibility to be adapted across different contexts, and hence contribute to ongoing discussions on standardization. Mistakes and errors are inevitable: such a model as the one described in this paper does not claim to eradicate them all, but to reduce their likelihood and severity by punctuating workflows with tailored checkpoints and safeguards. New caught inconsistencies get converted into new control points, increasing general “test coverage rate” (Miller and Maloney, 1963) across iterations, hence tending toward better global data quality assessment in the long run–provided no changes affect the data source.

We also think that such a model, by integrating a routine automatic collection of quality-related parameters, on one side, and a component for facilitated collaborative visual review, on the other, may efficiently serve as a stepping stone for improved automatic classifiers for QC and potentially contribute with new crowdsourced quality metrics, as proposed by Esteban et al. (2019a). Following this, one interesting future development would be to connect *snaprate* to MRIQC’s automatic prediction (Esteban et al., 2017).

On a different level, tools like *monitors* or *bx* are also based on XNAT, through calls to its REST API using *pyxnat*, and as such help in achieving customized and diversified user experience with the database.

We hence present a collection of basic individual components that, taken as a whole, form a novel ecological arrangement based on strong core principles (lightweight, reuse of existing tools, and reproducibility), which has shown efficiency in the context of single-site imaging cohort studies conducted by an individual research platform. Again, modularity makes it easy to take one or several components and allow their reuse by other groups, primarily the ones making use of large neuroimaging datasets for their research.

Finally, some of the presented components such as *snaprate* or *nisnap* are purely independent from XNAT since they are based on source-agnostic snapshots and as such may be used in any framework. The other ones are interfaced with the platform core using a unique library, *pyxnat* (Figure 5), therefore making the whole system virtually adaptable to other types of platforms just by replacing the binding module. Nevertheless, by leveraging its built-in features in particular for access right management, we believe that having XNAT as a cornerstone of the model is bound to have a downstream positive impact on data sharing (Herrick et al., 2016), primarily in groups lacking the necessary technical support (Poline et al., 2012; Haselgrove et al., 2014).

## Conclusion

Quality control of neuroimaging datasets and their processed derivatives is still an open problem in all cohort studies and generally synonymous with heavy burden. Its strong dependence on protocol specifications (i.e., study design, imaging protocol, and processing workflows) hinders the adoption of standardized approaches. Furthermore, the nature of subsequent analyses is also linked to the right verification procedure to implement and the same dataset may have to go through different QC passes depending on the final research question. To cope with this, a substantial amount of intermediate control steps may be automatized, as described in this paper, while the remaining needed visual inspection may be facilitated by integrated collaborative semi-automatic tools. As both aspects are tightly interconnected, all these QC procedures must be supported by some flexible and efficient data management strategies. We showed in that context that, capitalizing on existing components and by only adding some light interaction layer between them, user experience in accessing data can be diversified and thus fit with a variety of user profiles. Hence, providing improved access to data at its source is bound to give way to better analysis workflows in terms of traceability and reproducibility. All these components take part in a whole ecosystem that has been assembled and is currently running at the BBRC, an individual research unit managing cohort research programs on AD. By its modularity and the lightweight footprint/reusability of its parts, this ecosystem may be easily adjusted and/or augmented in accordance with other research groups’ needs.

## Members of the ALFA Study

The following are the collaborators of the ALFA study: Müge Akinci, Annabella Beteta, Raffaele Cacciaglia, Alba Cañas, Irene Cumplido, Carme Deulofeu, Ruth Dominguez, Maria Emilio, Karine Fauria, Sherezade Fuentes, Oriol Grau-Rivera, José M. González de Echevarri, Laura Hernandez, Gema Huesa, Iva Knezevic, Eider M. Arenaza-Urquijo, Eva M. Palacios, Paula Marne, Marta Milà-Alomà, Tania Menchón, Carolina Minguillon, Albina Polo, Sandra Pradas, Blanca Rodríguez, Aleix Sala Vila, Gemma Salvadó, Gonzalo Sánchez-Benavides, Mahnaz Shekari, Anna Soteras, Laura Stankeviciute, Marc Suárez-Calvet, Marc Vilanova, and Natalia Vilor-Tejedor.

## Data Availability Statement

The original contributions presented in the study are included in the article/Supplementary Material, further inquiries can be directed to the corresponding author/s.

## Ethics Statement

The ALFA study was approved by the Independent Ethics Committee “Parc de Salut Mar,” Barcelona. All participating subjects and signed the study’s informed consent form that had also been approved by the Independent Ethics Committee “Parc de Salut Mar”, Barcelona. The patients/participants provided their written informed consent to participate in this study.

## Author Contributions

JH co-implemented the ecosystem and co-wrote the manuscript. CF contributed to data acquisition and analysis and provided a critical revision of the manuscript. DF provided a critical revision of the manuscript. SG and DV provided expertise in high-performance computing and critical revision of the manuscript. JM and JG supported the design of the whole study, provided a critical revision of the manuscript, and supervised this project. GO designed and co-implemented the tools, supervised the development of the ecosystem, and wrote the manuscript. All authors read and approved the final manuscript.

## Conflict of Interest

JM is currently a full-time employee of Lundbeck and priorly has served as a consultant or at advisory boards for the following for-profit companies, or has given lectures in symposia sponsored by the following for-profit companies: Roche Diagnostics, Genentech, Novartis, Lundbeck, Oryzon, Biogen, Lilly, Janssen, Green Valley, MSD, Eisai, Alector, BioCross, GE Healthcare, and ProMIS Neurosciences. JG has received speaker’s fees from Biogen and Philips. The remaining authors declare that the research was conducted in the absence of any commercial or financial relationships that could be construed as a potential conflict of interest.

## Footnotes

1 http://www.bsc.es

2 As inventoried by the command: bx lists.

3 Adapted from Raymond (1999)

4 For instance, using the following bx command: bx spm12 report <project> (to download reports from SPM12 segmentations from a whole XNAT project).

5 The command would be: bx spm12 tests <project>.

6 https://gitlab.com/bbrc/xnat/bbrc-validator

7 http://github.com/xgrg/nisnap

8 https://github.com/rii-mango/Papaya

9 Using the command: bx snapshot <project>.

10 http://www.tornadoweb.org

11 https://incf.github.io/niQC/
